# The Influence of Focused Attention and Open Monitoring Mindfulness Meditation States on True and False Memory

**DOI:** 10.1007/s41465-023-00259-w

**Published:** 2023-03-30

**Authors:** Sara Bitton, Alex Chatburn, Maarten A. Immink

**Affiliations:** 1 University of South Australia, Adelaide, Australia; 2 Flinders University, Adelaide, Australia

**Keywords:** Focused attention meditation, Open monitoring meditation, Mindfulness, Attention, False memory, Memory, Cognitive control, Encoding, Retrieval, Deese-Roediger-McDermott (DRM) task

## Abstract

Previous investigations into the effect of mindfulness meditation on false memory have reported mixed findings. One potential issue is that mindfulness meditation involves different styles that establish distinct cognitive control states. The present work aimed to address this issue by comparing the effects of single-session focused attention (FAM) and open monitoring (OMM) mindfulness meditation styles on true and false memory recall. Strengthened cognitive control states associated with FAM were predicted to increase true memory recall and decrease false memory recall. Conversely, weakened cognitive control established by OMM was predicted to increase false memory recall. Thirty-four meditation-naïve participants (23 females, mean age = 23.4 years, range = 18–33) first completed pre-meditation learning and recall phases of the Deese-Roediger-McDermott (DRM) task. Participants then completed a single session of FAM or OMM prior to a second, post-meditation, round of DRM task learning and recall phases with a novel word list. Finally, participants completed a recognition test with true and false memory, and distractor words. Both FAM and OMM groups demonstrated significant increase in false memory recall between pre- and post-meditation recall tests but these groups did not differ with respect to true and false memory recall and recognition. The present findings are consistent with previous reports of increased false memory arising from mindfulness meditation. Distinct cognitive control states associated with FAM and OMM states do not result in distinct true and false memory formation, at least in meditation-naïve adults.

The purposeful allocation of attention to information is thought to be a critical determinant for memory formation and retrieval (Brown et al., 2016). Thus, it would be expected that attention augmentation leads to enhanced memory. One such attention augmentation approach has involved mindfulness meditation. Although mindfulness meditation techniques are often associated with relaxation and stress management, recent work has demonstrated their potential to influence a range of cognitive processes, such as attention, memory, executive function and visuo-spatial processing (Brown et al., 2016; Colzato et al., 2015a; Zeidan et al., 2010). Given that mindfulness meditation has been described as an awareness that arises from the purposeful directing of attention to the present moment in an accepting and non-judgemental fashion (Kabat-Zinn, 2003), meditation is thought to influence cognition through enhanced attentional control (Brefczynski-Lewis et al., 2007; Hölzel et al., 2011).

Current theoretical descriptions of mindfulness meditation place attentional control as a core process of mindfulness techniques (Bishop et al., 2004; Hölzel et al., 2011; Lutz et al., 2008; Malinowski et al., 2013; Moore et al., 2012; Moore & Malinowski, 2009). Attention control enables the practitioner to focus and maintain attention on the meditation object — body sensations, breathing or thoughts, for example — and to redirect attention back to the object of the meditation when mind-wandering occurs. Not surprisingly then, studies have found that mindfulness meditation benefits a range of attention control-related components including sustained attention (Valentine & Sweet, 1999; Zeidan et al., 2010), attentional orienting (Jha et al., 2007), selective attention (Colzato et al., 2016), cognitive inhibition (Chan et al., 2017; Tang et al., 2007) and cognitive flexibility (Colzato et al., 2015a). For example, Chan et al. (2020), using EEG event-related potential methodology during motor sequence learning, reported heightened N2 amplitude, a neurophysiological marker of increased attention regulation, after single-session meditation when it was preceded by brief mindfulness meditation training. Thus, a base of empirical evidence supports enhanced attentional control processes as underlying cognitive enhancement from mindfulness meditation (Lippelt et al., 2014).

Mindfulness training has been found to benefit both encoding and retrieval processes leading to increased source memory retrieval (Nyhus et al., 2019), recognition memory (Basso et al., 2019; Brown et al., 2016), autobiographical memory specificity (Heeren et al., 2009) and free recall (Brown et al., 2016; Lykins et al., 2012). Mindfulness meditation has also been found to benefit higher-order memory processes (Brown et al., 2016; Lykins et al., 2012; Nyhus et al., 2019). For instance, compared to controls, brief training in focused attention meditation has been shown to enhance performance in episodic memory tasks (Brown et al., 2016). Performance benefits in long-term memory functioning have also been found in experienced meditators (Lykins et al., 2012) who demonstrate long-lasting changes in hippocampal functioning, an area of the brain crucial to memory performance (Lardone et al., 2018).

In addition to enhanced memory for previously experienced information, mindfulness meditation has also been shown to increase false memory (Rosenstreich, 2016; Wilson et al., 2015). False memory refers to a memory generalisation phenomenon that occurs when an individual recalls or recognises a critical item as a studied item even though the critical item was not previously studied. Two theoretical explanations have been proposed to describe false memory formation. According to Activation Monitoring Theory (Gallo, 2010; Gallo & Roediger, 2002; Roediger & McDermott, 1995), false memory first arises from the encoding stage because studied items activate a semantically associated critical item. Then at retrieval, monitoring processes do not detect the critical item as exclusive to the studied items and therefore, the critical item is not rejected (Gallo, 2010). Fuzzy-Trace Theory (Brainerd & Reyna, 2002), on the other hand, describes false memories as arising two types of memory representation. Semantic and conceptual information is represented as the gist, whilst specific perceptual and contextual information is represented as the verbatim. Retrieval reliance on the gist, as opposed to the verbatim, results in low discriminability between studied and critical items.

Wilson et al. (2015) conducted a series of experiments exploring the effects of mindfulness meditation on false memory formation in the Deese-Roediger-McDermott (DRM) paradigm (Roediger & McDermott, 1995). Their findings indicated that participants in a mindfulness meditation condition were significantly more likely than those in a mind-wandering control to falsely recall words never studied in the DRM, and to incorrectly judge non-studied words as previously learned in a separate word-association task. These findings were supported by Rosenstreich (2016) who reported that a mindfulness training group falsely recognised significantly more words on the DRM than a waitlist control. In addition, these studies observed a significant increase in false memories from pre- to post-meditation. Increased false memory incidence from mindfulness meditation was attributed to mindfulness meditation influences on retrieval processes, either by weakening cognitive control of monitoring mechanisms (Wilson et al., 2015) or increasing reliance on semantic associations as opposed to specific information (Rosenstreich & Ruderman, 2017). However, in contrast to Wilson et al., (2015), Rosenstreich (2016) reported increased accuracy for recognition of studied items following mindfulness meditation. Thus, it was apparent that mindfulness meditation can increase false and true memory rates. This contradiction was explained as occurring when reduced attention allocation at encoding reduces semantic activation of the critical item together with mindfulness meditation increasing reliance on semantic level information at the recall stage (Ayache et al., 2022; Knott & Dewhurst, 2007).

The effects of mindfulness meditation on increased false memory have been difficult to reproduce with work also reporting either decreased (Baranski & Was, 2017; Calvillo et al., 2018) or no difference in false memory incidence (Ayache et al., 2022; Baranski & Was, 2017; Meeks et al., 2019; Sherman & Grange, 2020) from mindfulness meditation. Divergence in reported findings might arise from inconsistent methodologies employed in this research. For example, studies have differed with respect to whether mindfulness meditation was experience before (Ayache et al., 2022; Meeks et al., 2019; Rosenstreich, 2016; Sherman & Grange, 2020; Wilson et al., 2015) or after (Baranski & Was, 2017; Calvillo et al., 2018). Another potential issue might relate to the use of mind-wandering as a control condition (e.g. Baranski & Was, 2017; Rosenstreich, 2016; Wilson et al., 2015). In question is whether mind-wandering is an appropriate control for meditation given Sherman and Grange (2020) demonstrated overlapping processes between mind-wandering and mindfulness meditation. A third example of methodological inconsistency relates to instances where participants received warnings that tests included memory lures prior to memory retrieval tests (Baranski & Was, 2017; Wilson et al., 2015).

Confounds between trait mindfulness and mindfulness state (Wheeler et al., 2016) in false memory research might be another source for mixed findings. Trait, or dispositional, mindfulness relates to an intrinsic and enduring expression of mindfulness that does not require one to be actively engaged in a meditation technique whereas mindfulness state refers to the intentional and more temporary perceptual, cognitive or behavioural features or experiences established by a meditation technique (Cahn & Polich, 2006; Tang et al., 2016). Whilst most false memory studies have involved brief, single-session mindfulness meditation, which would relate to mindfulness state effects on false memory, in Rosenstreich (2016, Experiment 1), participants in the mindfulness meditation group completed 5 weeks of mindfulness training prior to encoding, where meditation training might have contributed to changes in trait mindfulness. Since high levels of trait mindfulness have been associated with increased true and false memory formation (Ayache et al., 2022; Yeh & Lu, 2017), trait mindfulness needs to be considered as an individual difference that independently influences true and false memory recall. Individual differences in trait mindfulness might also influence the extent to which a mindfulness state is established by a meditation technique resulting in disparate effects the meditation technique on false memory. These points are especially noteworthy given that formal mindfulness training is not necessary to establish individual differences in train mindfulness (Brown & Ryan, 2003).

As mindfulness meditation refers to a wide range of techniques, another prevailing issue in the reported mixed findings likely involves heterogeneity in the mindfulness techniques investigated. Accordingly, there have been specific calls for research work to provide specific details about the mindfulness meditation technique being investigated (Lutz et al., 2008). As an example of this issue, two previous studies (Baranski & Was, 2017; Wilson et al., 2015) included a focused-attention meditation (FAM) style of mindfulness meditation. In contrast, three other meditation and memory generalisation studies (Calvillo et al., 2018; Meeks et al., 2019; Rosenstreich, 2016) involved a technique that combined the FAM style with another mindfulness meditation style termed open-monitoring meditation (OMM). In summary, the mixed results reported in memory generalisation following mindfulness meditation states might reflect a confound in how FAM and OMM styles distinctly shape attention.

Attention control has been described as a primary characteristic that distinguishes FAM and OMM mindfulness meditation styles (Immink et al., 2017; Lippelt et al., 2014; Lutz et al., 2008). The goal of FAM is to focus and maintain attention on an explicit object or target such as the breath (Immink et al., 2017; Lutz et al., 2008). The selective nature of the FAM goal relies on increased executive control of attention to narrow focus and increase competition with distracting information (Chan et al., 2017, 2020; Colzato et al., 2015a, 2016; Lippelt et al., 2014). Consistent with this idea, FAM has been found to increase attention control on several cognitive tasks such as the Symbol Digit Modalities test, verbal fluency, n-back (Zeidan et al., 2010), attention orienting (Jha et al., 2007) and the Wilkins’ counting test (Valentine & Sweet, 1999). More specifically, Colzato et al., (2016) reported that meditation naïve participants demonstrate increased ability to supress task-irrelevant visual stimuli on the global–local task after a brief 17-min session of FAM.

Whilst FAM increases attention selectivity, OMM is thought to engender more inclusive attention states (Colzato et al., 2015a). The goal of OMM is to maintain attention of several simultaneous objects or experiences, for example — breathing, body sensations, sounds and thoughts (Colzato et al., 2012; Immink et al., 2017; Lippelt et al., 2014; Lutz et al., 2008). To render a broader scope of attention, whereby there is less competition between concurrent information, executive regulation must be weakened. This was demonstrated by Colzato and colleagues (2016) who found that compared to FAM, engaging in a brief session of OMM led to a considerably larger congruency effect in the global–local task. This indicates that the cognitive state facilitated by OMM leads to greater difficulty in supressing task irrelevant information. Similarly, meditation experience with OMM results in higher performance than FAM meditation experience in tasks involving unexpected target stimuli (Valentine & Sweet, 1999). This supports the notion that OMM promotes a more encompassing, divergent thinking style that weakens top-down control (Colzato et al., 2012). Moreover, a single-session of OMM results in heightened adaptation following a trial with incongruent information in the Simon task (Colzato et al., 2015b). These findings illustrate that in contrast to FAM, OMM establishes a more flexible and less selective attentional state.

Contemporary theoretical descriptions of mindfulness meditation have highlighted the role of cognitive control as a primary driver of attentional deployment in mindfulness techniques (Chang et al., 2018; Chiesa et al., 2011; Gallant, 2016; Moore & Malinowski, 2009; Moore et al., 2012). Cognitive control, also referred to as executive control, ensures maintenance of goal-oriented behaviour by attuning attention to goal-relevant information and inhibiting irrelevant or distracting information (Morton et al., 2011). Cognitive control itself has been proposed to be regulated by the metacontrol policy that best serves goal behaviour (Hommel, 2015). Specific to mindfulness meditation, the metacontrol state model (Hommel & Colzato, 2017) describes the narrow, selective attention state established by FAM as arising from increased cognitive control, which arises from a persistence metacontrol policy. During FAM, a persistence metacontrol policy ensures that narrowed attention is sustained on information related to the technique’s single object or experience and in the event of distraction, that attention is redirected to the goal-relevant information. In contrast, during OMM, a flexible metacontrol policy weakens cognitive control to allow multiple information sources to be attended to simultaneously.

According to the metacontrol state model, the cognitive control state established during mindfulness meditation is relatively inert (Hommel & Colzato, 2017). This means that behaviour subsequent to completion of the mindfulness technique continues to be shaped by the metacontrol policy established during the mindfulness state (Colzato et al., 2015a; Lippelt et al., 2014). Moreover, because mindfulness techniques employ available cognitive control processes, the influence of mindfulness meditation on subsequent behaviour does not require training with the technique (Colzato et al., 2015a; Lippelt et al., 2014). For example, compared to OMM, a single session of FAM, with meditation naïve participants, has been found to subsequently increase the attentional blink response (Colzato et al., 2015a). Failure of FAM participants to accurately detect two targets when presented in close succession indicates that a single session of FAM leads to an immediate increase in the cognitive control state promoting serial processing and the narrowing of attention. Supporting this notion, Ullrich et al., (2021) found that a single session of FAM with novice meditators was sufficient to bias the cognitive state towards persistence as indexed by the limited retrieval of stimulus–response bindings to relevant information in the event file task. Similarly, a single session of OMM prior to the completion of the Simon task led to an increase in adaptations to previous conflict (Colzato et al., 2015b). This indicates that the cognitive control states induced by meditation persist beyond the meditation session (Immink et al., 2017).

The instantaneous effects that meditation states exert on cognitive control have also been found to influence the domain of memory. For example, FAM has been found to immediately enhance the cognitive control state leading to an increase in stimulus–response processing (Chan et al., 2018) and to bias the implementation of stimulus-based planning leading to improvements in motor sequence learning (Chan et al., 2018). Evidence backing this idea comes from Chan et al. (2020) who reported an increased N2 event-related potential, reflecting increased cognitive control, during sequence learning immediately following FAM. In contrast, OMM has been shown to exert instantaneous improvements in the degree of sequence-specific learning (Immink et al., 2017). Therefore, due to its lasting effects, meditation research has the potential to further scientific understanding of the mechanisms that may enhance or impair cognition. In summary, FAM and OMM meditation styles are thought to establish opposing cognitive control states resulting in distinct effects on subsequent behaviour. As such, mixed use of FAM and OMM in previous false memory investigations might offer some explanation for the disparate findings in the mindfulness meditation and false memory literature. Specifically, strengthened cognitive control and narrowed attention associated with FAM might promote heightened true memory recall whilst reducing memory generalisation. Indeed Brown et al. (2016) reported enhanced memory recognition and recall when a 10-min session of FAM preceded encoding. Conversely, weakening of cognitive control and broadened attention states arising from OMM might result in increased memory generalisation and therefore, greater incidence of false memory.

The purpose of the current study was to investigate how the distinct cognitive control states established by different mindfulness meditation styles may extend to influence other cognitive operations, such as memory. The current experiment was designed to compare the effects of FAM and OMM mindfulness meditation styles on the formation of true and generalised, or false, memory. This experiment offered the opportunity to address current discrepancies in reported effects of mindfulness meditation on declarative memory formation. In addition, we sought to extend previous work on the effects of FAM and OMM on subsequent behaviour (e.g. Colzato et al., 2015a; Lippelt et al., 2014) by inspecting the immediate effects of these mindfulness techniques on memory to complement the previous focus on attention processes. Immediate retroactive effects of FAM and OMM mindfulness styles on true and false memory at encoding by treating FAM and OMM mindfulness meditation as a between-subject factor. Furthermore, a repeated measures design was employed to compare true and false memory when encoding was preceded by control conditions versus single-session mindfulness meditation.

## Materials and Methods

### Participants

Thirty-four adults (23 females, 23. 4 ± 3.3 years) participated in the present Experiment. A priori sample size estimate of 34 participants was calculated in G*Power (Version 3.1.9.7; Faul et al., 2007) to detect a medium effect size of *f* = 0.25 based on a between-groups, repeated measures design with a 0.05 significance criterion level and 0.80 power.

All recruitment material and participant information advertised the study as being an investigation of cognitive states and memory. To reduce potential expectation bias, the meditation was described as an auditory cognitive task. Participant inclusion criteria included between 18 and 35 years of age, inclusive, proficient in spoken English, normal vision (with or without corrective lenses/glasses) and hearing (with or without hearing aids) and no prior formal or cognitive training including meditation or mental training. The protocol for this research was approved by the University of South Australia Human Research Ethics Committee and individuals provided written informed consent prior to participation.

Following consent, participants were randomly allocated to either a FAM (*N* = 16, 10 females, 6 males) or OMM (*N* = 18, 13 females, 5 males) meditation group within male and female gender blocks to ensure equivalent distribution of males and females. Randomised group allocation lists were generated for males and females and each participant was allocated on a rolling basis. Participants reported whether they had a history of sleep difficulties (e.g. insomnia; *N* = 3; 2 FAM, 1 OMM), drug or alcohol dependence (*N* = 2; 1 FAM, 1 OMM), attention, cognitive impairments or psychiatric diagnoses (*N* = 3; 1 FAM, 2 OMM) and intellectual impairments (*N* = 0).

### Measures

#### Mindful Attention Awareness Scale (MAAS)

The MAAS is a 15-item self-report scale designed to measure a core characteristic of trait mindfulness, namely receptive awareness or attention to the present moment (Brown & Ryan, 2003). The MAAS was included in the protocol as a check that participant allocation did not result in group differences for mindfulness disposition. If differences were detected, MAAS scores were to be included in the analysis as a covariate. MAAS scores were also evaluated for potential correlation with true and false memory recall and recognition. The instrument was completed by participants in an online survey format and took approximately 5 min. All items were rated from 1 (almost always) to 6 (almost never), with a total score ranging from 15 to 90, with higher scores reflecting greater trait mindfulness (Brown & Ryan, 2003). Scores were then divided by the number of questions (15) to determine the participant’s item average. For example, a total score of 35 on the MAAS resulted in an item average of 2.3. Item averages range from 1 to 6.

#### The Deese-Roediger-McDermott (DRM) Task

The DRM is a word-learning task designed to provoke and test the formation of semantic-associative false memories (Roediger & McDermott, 1995). The current work employed a modified DRM paradigm. Although the typical DRM paradigm consists of 12–15 word-learning lists, 6 lists (see Table 1) were utilised for the current experiment (Roediger & McDermott, 1995). Each list consisted of 15 words (study items) and 1 critical lure item which was not presented (Roediger & McDermott, 1995). Based on standard DRM protocols (e.g. Gallo & Roediger, 2002; Pardilla-Delgado & Payne, 2017; Roediger & McDermott, 1995), words from each list were ordered in decreasing relatedness to the critical lure.

**Table 1:** A and B sets of DRM word lists utilised in the present experiment. Recall of the critical (non-presented) item reflects false memory

*Critical item*	Set A			Set B		
	*Chair*	*Smell*	*Window*	*Doctor*	*Sleep*	*Sweet*
Studied items	Table	Nose	Door	Nurse	Bed	Sour
	Sit	Breathe	Glass	Sick	Rest	Candy
	Legs	Sniff	Pane	Lawyer	Awake	Sugar
	Seat	Aroma	Shade	Medicine	Tired	Bitter
	Couch	Hear	Ledge	Health	Dream	Good
	Desk	See	Sill	Hospital	Wake	Taste
	Recliner	Nostril	House	Dentist	Snooze	Tooth
	Sofa	Whiff	Open	Physician	Blanket	Nice
	Wood	Scent	Curtain	Ill	Doze	Honey
	Cushion	Reek	Frame	Patient	Slumber	Soda
	Swivel	Stench	View	Office	Snore	Chocolate
	Stool	Fragrance	Breeze	Stethoscope	Nap	Heart
	Sitting	Perfume	Sash	Surgeon	Peace	Cake
	Rocking	Salts	Screen	Clinic	Yawn	Tart
	Bench	Rose	Shutter	Cure	Drowsy	Pie

Recall of a studied item reflects true memory. Set A and B lists were studied separately in pre- and post-meditation learning phases with the order of word list sets counterbalanced between participants. Word lists are from Roediger et al. (2001)

Whilst previous work addressing mindfulness meditation influences on false memory have employed memory retrieval tasks involving recognition (Baranski & Was, 2017) or recognition and recall (Ayache et al., 2022; Rosenstreich, 2016; Sherman & Grange, 2020; Wilson et al., 2015) tests, in the present work, we assessed true and false memory retrieval based on recall and recognition tests as false memory in the DRM task has been shown to be robust under recall and recognition retrieval conditions (Coburn et al., 2021).

### Procedure

Due to the COVID-19 pandemic and associated social distancing health requirements and state-wide lockdowns precluding in-person contact, the experiment was conducted remotely. Experiment scripts were generated in E-Prime 3 and packaged in E-Prime Go (Psychological Software Tools Inc., Sharpsburg, PA). Four versions of the experiment were created — 2 including FAM and 2 with OMM but with counterbalanced orders for DRM word list sets in pre- and post-meditation phases. Individuals who were interested in participating contacted the researcher via email. Eligible participants were required to electronically sign and return the consent form before being randomly allocated to the FAM or OMM group. The participant was then emailed the link to the online survey, the experiment instructions and the link for the script corresponding to their group allocation.

#### Online Survey

**Fig. 1:**
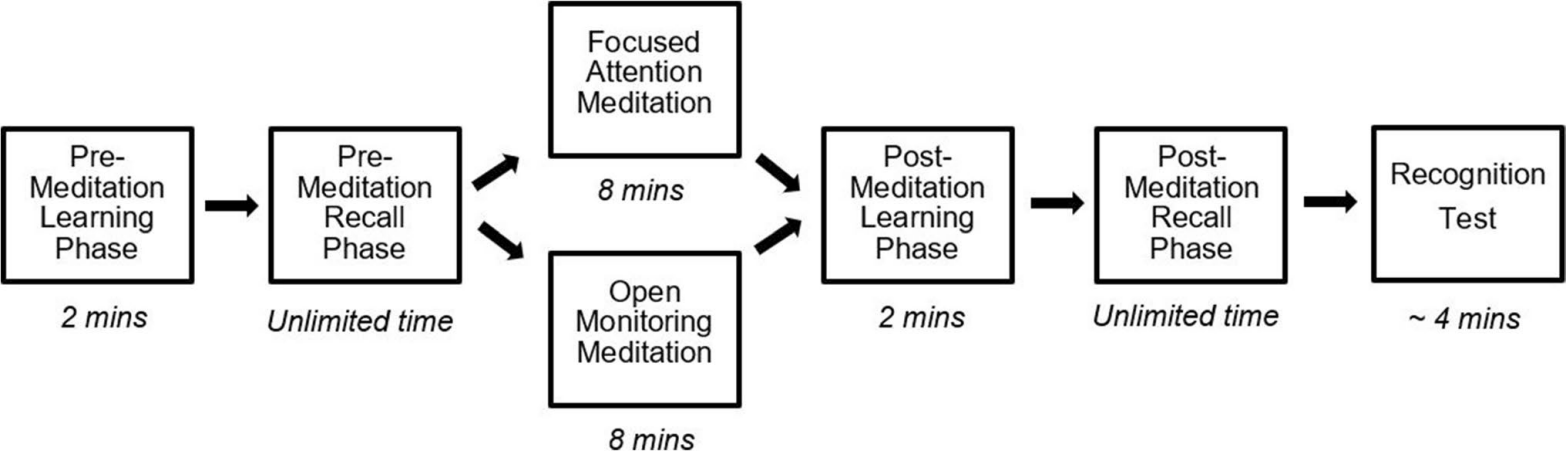
Experiment procedure

Participants followed a link to LimeSurvey where they answered demographic questions, including their age and gender, history of sleep difficulties, drug or alcohol dependence, cognitive, attention or psychiatric diagnosis and intellectual impairments. Participants then completed the MAAS. After completing the 5-min survey, participants followed a separate emailed link to download the E-prime Go experiment script, which ran on the participant’s computer. The procedure for the remote experiment is presented in Fig. 1.

#### Pre-meditation Learning Phase

**Fig. 2:**
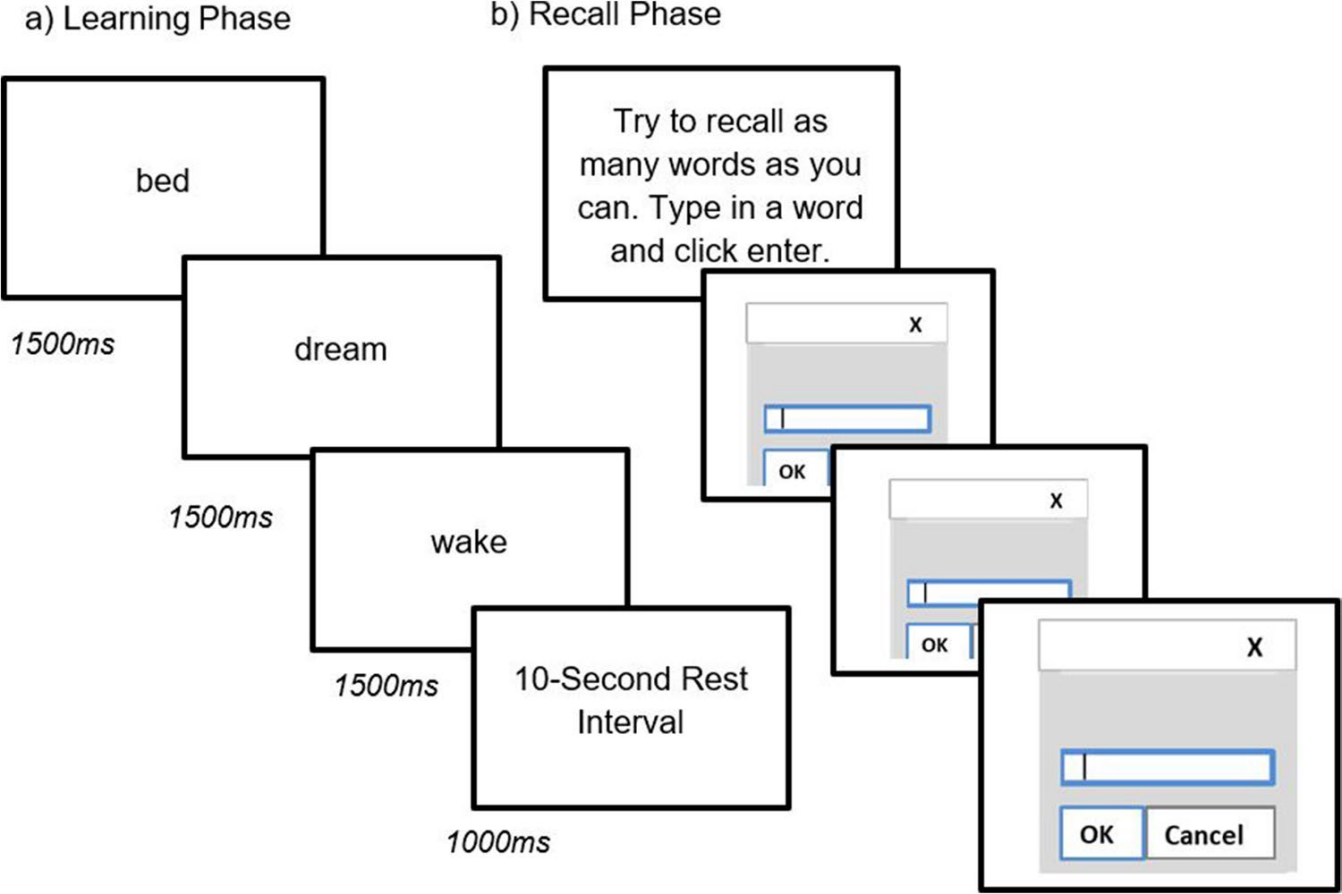
DRM task example. A learning phase involved sequential presentation of words from lists that were semantically related to a critical lure word. The recall phase required participants to recall and enter as many words as they could remember into the text box provided. Words were entered one at a time

The experiment began with the first, pre-meditation, learning phase of the DRM paradigm. During the learning phase, 3 lists comprising of 15 words each were presented on the screen sequentially for 1500 ms with a 10-s rest interval between lists (see Fig. 2). Participants were exposed to 3 DRM lists from Roediger et al. (2001) at pre-meditation (Table 1, set A) and 3 lists post-meditation (Table 1, set B), depending on their allocated world list set order*.* The presentation of sets A and B was counterbalanced to account for order effects. Participants were instructed to commit words to memory as best they could.

#### Pre-meditation Recall Phase

Immediately following the learning phase, participants began the pre-meditation recall phase of the experiment which required participants to recall as many words from the learning phase as they could. Participants were instructed to type, one at a time and in any order, all the words that they recalled studying (see Fig. 2).

#### Mindfulness Induction

Following the pre-meditation learning and recall phase, participants completed an 8-min session of either FAM or OMM. The meditation was described to participants as an auditory cognitive task. In both conditions, a pre-recorded audio, presented as part of the E-Prime Go experiment script, was presented in English through headphones connected to the participant’s personal computer. Participants were instructed to remain seated for the task, to close their eyes, to refrain from moving and sleeping and to follow the instructions of the audio-guided exercise as best as possible. The instructions for FAM and OMM were based on transcripts that were previously used by Immink et al. (2017) and have been found to influence cognitive control (Colzato et al., 2015a). In both conditions, an accredited male meditation teacher voice guided participants through the meditation. Following completion of their respective meditation session, participants completed ratings on their meditation experience related to perceived effort, motivation for and success in completing the mindfulness technique. Ratings were obtained based on a sliding scale on a 100-point visual analogue scale (VAS), with the left anchor text, “None”, and the right anchor text, “Maximum”. For meditation Effort, participants were asked to rate, “How much effort was required to complete the cognitive auditory task?”. For meditation Motivation, participants were asked to rate, “How motivated were you to complete the cognitive auditory task?”. For meditation Success, participants were asked to rate, “How successful were you in completing the cognitive auditory task?”.

Participants in the FAM group were guided step-by-step to focus and sustain attention on their breathing. If mind wandering occurred, participants were instructed to return attention to the breath. In the OMM group, participants were guided step-by-step to monitor their awareness of their breathing, thoughts, feelings and bodily sensations from moment-to-moment without judgement or emotional reactivity.

#### Post-meditation Learning Phase

Immediately after, participants began another, alternate learning phase of the DRM paradigm. Other than the presentation of a separate set of word lists, the second learning phase was identical to the pre-meditation learning phase.

#### Post-meditation Recall Phase

This phase involved the same conditions as the pre-meditation recall phase. In total, the online experiment took approximately 20 min.

#### Recognition Test

The recognition tests consisted of 120 trials where a true memory, false memory or distractor word was presented individually, and participants had a two-choice forced response to indicate if the word was previously studied in pre- and post-meditation lists or not. The trials consisted of 90 studied words (true memory) from pre- and post-meditation word lists, 6 critical item words (false memory) from pre- and post-meditation word list sets, and 24 distractor words, which were non-studied words from lists described by Roediger et al. (2001). Recognition test performance was based on accuracy and reaction time. A correct response was when the participant responded to a true memory word as being previously studied or responded to a false memory word or a distractor word as not being previously studied. Reaction time was calculated as the latency between word appearance and response entry. Rationale for the inclusion of recognition reaction time performance was to assess if recognition accuracy was influenced by a potential trade-off between latency of responding and accuracy the response. No feedback was provided after responses and a 1-s interval intervened word presentations.

### Data Analysis

Data analysis was conducted in IBM SPSS Statistics for Windows, Version 27. Separate independent samples *t*-tests were conducted assess if the mindfulness meditation groups significantly differed in terms of participant age and mindfulness disposition, the latter based on MAAS scores. Age and MAAS data was not provided by one participant in the OMM group. Chi-square tests were conducted to test for group differences in gender distribution and the distribution of DRM word list order between pre- and post-meditation. Additional independent samples *t*-tests were conducted to test for group differences in ratings of meditation session related to effort, motivation and perceived success. Pearson correlation coefficients were calculated between participant MAAS scores and meditation ratings.

For each participant and recall test (i.e. pre- and post-meditation), recall percentage was calculated for true memory (studied items) and false memory (critical items). Participant recall percentages underwent outlier detection. Although outlier detection identified two high values (100%) in the FAM group and two high values (100%) in the OMM group for false memory recall percentage at pre-meditation and two high values (62.2%, 71.1%) in the FAM group for true memory recall at post-meditation, these values were considered to reasonably reflect recall percentage performance and thus, meaningful for the present purpose. Therefore, these values were not excluded from inferential analysis. Data were then analysed for normality. The Shapiro–Wilk test was used as is it is deemed appropriate for small sample sizes (Le Boedec, 2016). All data were normally distributed except for the false memory recall percentage variable which violated normality (*p* = 0.003). However, skew and kurtosis for false memory recall percentage did not breach the cut-off score of 2 and thus were considered within acceptable limits of robustness for an analysis of variance (ANOVA; Field, 2009). Therefore, all recall percentage data underwent parametric inferential analysis. Recall percentage was submitted a 2 (Group: FAM, OMM) × 2 (List Order: A-B, B-A) × 2 (Memory Type: True, False) × 2 (Test Time Point: Pre- and Post-meditation) ANOVA with repeated measures on the latter two factors. The List Order factor reflected whether participants studied word list sets A or B at pre-meditation recall and then the other word list at post-meditation recall (i.e. Orders AB or BA) (see Table 1 for word lists under sets A and B). If participant age, MAAS scores or meditation rating VAS scores significantly differ between groups, these were to be included as covariates in an analysis of covariance using the factors outlined above. The locus of any significant interactions was evaluated with post hoc simple main effect analysis using least significant difference (LSD).

To test if heightened true and false memory recall was associated with trait mindfulness (Ayache et al., 2022; Yeh & Lu, 2017), Pearson correlation coefficients were calculated between MAAS scores, and pre- and post-meditation true and false memory recall. We also included meditation effort, success and motivation ratings in Pearson correlation coefficients calculations, based on the notion that perceived effort and self-efficacy, or success, in completion of the meditation technique might indirectly influence true and false memory recall. For example, higher effort ratings associated with completing FAM have been associated with higher mindfulness state effects on inhibitory control (Yamaya et al., 2023), whilst higher effort ratings for OMM have been associated with greater state effects on motor sequence learning (Immink et al., 2017). We correlated self-reported meditation motivation with true and false memory because motivation has been reported to reflect the quality of the meditation state (Spanos et al., 1980). Pre- and post-meditation count of recalled words not appearing in the studied lists or representing word list critical items was compared between FAM and OMM groups using generalised linear regression modelling with a Poisson distribution and Wald chi-square test.

For each participant, recognition test percent accuracy and mean reaction time was calculated according to word type (true memory, false memory, distractor) and word list study phase (pre-meditation, post-meditation). The word list factor was included in the analysis to test if recognition accuracy or reaction time dependent on the word being presented before or after the single-session meditation. Recognition accuracy and mean reaction time for true and false memory words were separately submitted to 2 (Group: FAM, OMM) × 2 (List Order: A-B, B-A) × 2 (Memory Type: True, False) × 2 (Test Time Point: Pre- and Post-meditation) ANOVA with repeated measures on the latter two factors. Recognition accuracy and mean reaction time for distractor words were separately submitted to 2 (Group: FAM, OMM) × 2 (List Order: A-B, B-A) ANOVA. MAAS scores or meditation rating VAS scores were to be included as covariates in the event of significant meditation group differences in these measures. The locus of any significant interactions was evaluated with post hoc simple main effect analysis using least significant difference (LSD). Pearson correlation coefficients were calculated between MAAS scores, meditation VAS ratings and pre- and post-meditation true and false memory and distractor word recognition accuracy and reaction time. The motivation for conducting these correlation analyses was similar to that described for true and false memory recall.

## Results

### Tests for Group Differences: Participant Characteristics, Meditation Ratings and DRM List Order

Mindfulness meditation style groups did not significantly differ in terms of gender distribution (*p* = 0.54), participant age (*p* = 0.52) or MAAS score (*p* = 0.24) (see Table 2). MAAS scores in the present sample, 3.75, are comparable to the mean score of 3.97 reported by Brown and Ryan (2003) for non-meditating community adults. The present sample is below the mean score of 4.38 reported for active (Brown & Ryan, 2003), supporting the notion that the current sample were naïve mindfulness meditators. Meditation VAS scores for Effort (*p* = 0.60), Motivation (*p* = 0.42) and Success (*p* = 0.38) did not significantly differ between FAM and OMM (see Table 2). MAAS scores were not significantly correlated with meditation VAS ratings for Effort (*p* = 0.08) or Motivation (*p* = 0.14) but were significantly correlated with meditation Success ratings, *r*(33) = 0.44, *p* = 0.01. Groups did not significantly differ with respect to the distribution of DRM word list order between pre- and post-meditation (*p* = 0.49) (see Table 2).

**Table 2:** Participant characteristics, meditation rating visual analogue scores (VAS) and Deese-Roediger-McDermott (DRM) task word list order for focused attention meditation and open monitoring meditation groups, and the overall sample

	Focused attention meditation	Open monitoring meditation		Overall
Characteristics				
* N*	16	18		34
Gender	10 F, 6 M	13 F, 5 M	*p* = .54	23 F, 11 M
Age (yrs)	23.8 (4.0)	23.1 (2.7)	*p* = .52	23.4 (3.3)
MAAS	3.55 (1.1)	3.95 (0.8)	*p* = .24	3.75 (1.0)
Meditation VAS				
Effort	46.6 (31.3)	40.9 (31.1)	*p* = .60	43.6 (30.9)
Motivation	68.9 (24.6)	76.0 (25.9)	*p* = .42	72.7 (25.2)
Success	67.6 (23.8)	59.7 (28.1)	*p* = .38	63.4 (26.1)
DRM task				
List order	9 AB, 7 BA	8 AB, 10 BA	*p* = .49	17 AB, 17 BA

Group and sample size, gender and DRM list order are presented as count data. All other data are presented as mean and standard deviation (SD). MAAS is the Mindful Attention Awareness Scale (Brown & Ryan, 2003). DRM list order AB, for example, represents study of list A at pre-meditation and list B at post-meditation phases

### Analysis of True and False Memory Recall

**Fig. 3:**
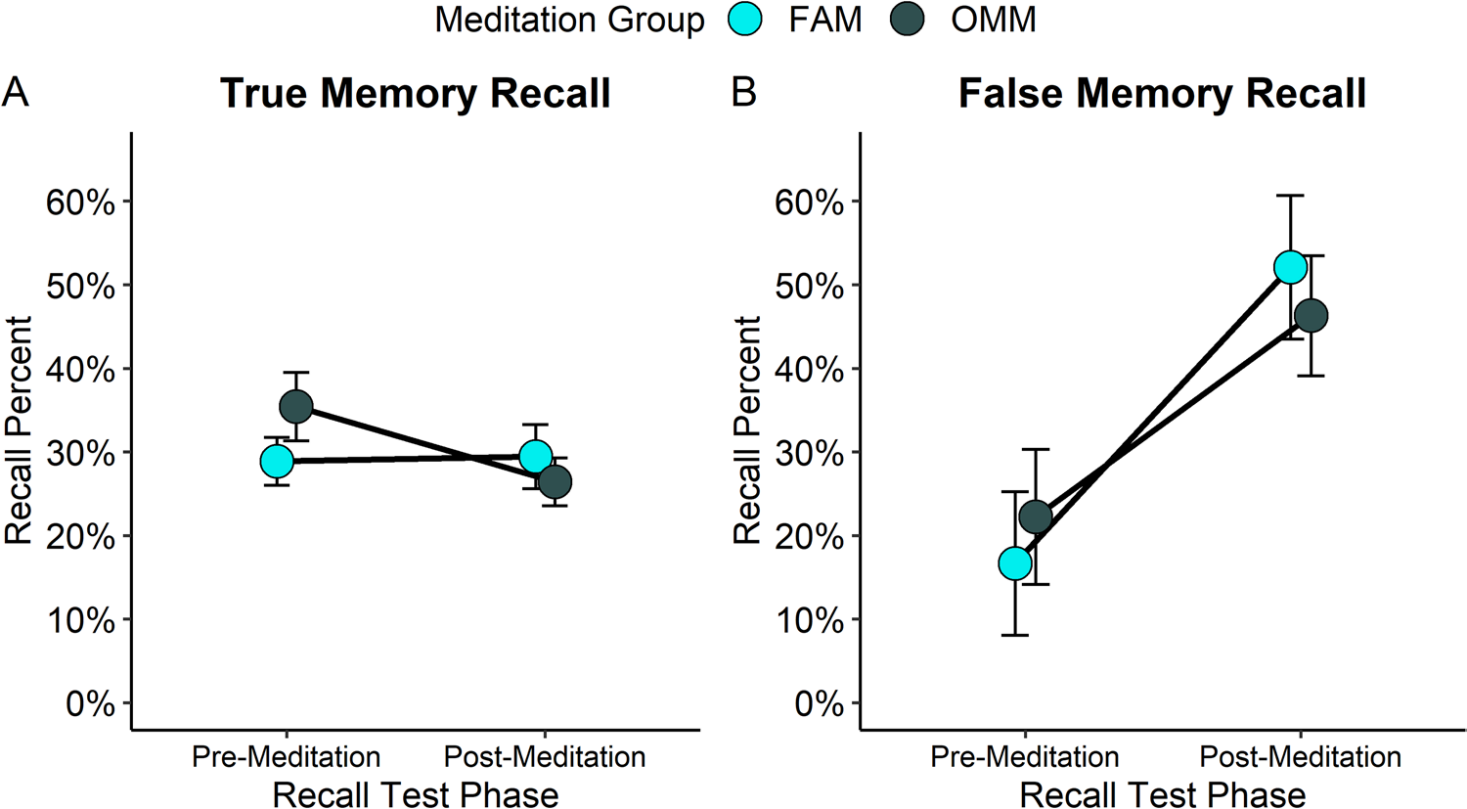
True (**A**) and false (**B**) memory recall percentage performance from pre- and post-meditation learning and recall phases for focused attention meditation (FAM) group and open monitoring meditation (OMM) groups. Pre-meditation learning and recall was not preceded by a session of meditation whilst single-session FAM or OMM preceded post-meditation learning and recall. Error bars represent standard error of the mean

Analysis of recall percentage revealed significant main effects of Memory Type (*F*[1, 30] = 14.08, *p* < 0.001, *η*^*2*^_partial_ = . 32) and List Order (*F*[1, 30] = 6.10, *p* = 0.019, *η*^*2*^_partial_ = 0.17. There was a significant 2-way interaction of Memory Type and Test Time Point (*F*[1, 30] = 18.00, *p* < 0.001, *η*^*2*^_partial_ = 0.382) but this was superseded by a significant Memory Type × Test Time Point × List Order interaction, *F*(1, 30) = 6.91, *p* = 0.013, *η*^*2*^_partial_ = 0.19. All other main effects and interactions were not significant. Simple main effects analysis for the significant 3-way interaction revealed that for true memory type, there was no significant difference in recall percentage between pre- (Mean = 32.75%, SE = 3.72) and post-meditation (Mean = 33.04%, SE = 3.20, *p* = 0.94) for the AB list order group. For the BA list order group, true memory recall at pre-meditation (Mean = 31.57%, SE = 3.77) was significantly higher than at post-meditation (Mean = 22.84%, SE = 3.24, *p* = 0.041). True memory recall for the AB group was significantly higher than the BA group at post-meditation (*p* = 0.033) but not pre-meditation (*p* = 0.83). For false memory recall, the AB list order group did not exhibit significant differences between pre- (Mean = 37.73%, SE = 7.19) and post-meditation (Mean = 50.93%, SE = 8.10, *p* = 0.18) tests. The BA list order group demonstrated significantly higher true memory recall at post-meditation (Mean = 47.88%, SE = 8.22) than pre-meditation (Mean = 1.67%, SE = 7.30, *p* < 0.001) tests. The AB list order group demonstrated significantly higher false memory recall at pre-meditation (*p* < 0.001). No significant list order group differences were observed for post-meditation false memory recall (*p* = 0.79). True and false memory mean recall percentages for FAM and OMM groups at pre- and post-meditation retrieval tests are presented in Table 3 and illustrated in Fig. 3.

**Table 3:** Recall percentages for true and false memory at pre-meditation and post-meditation for focused attention meditation (FAM) and open monitoring meditation (OMM) groups and meditation groups under DRM word list order groups

	Mindfulness meditation groups			
	True memory		False memory	
	Pre-meditation	Post-meditation	Pre-meditation	Post-meditation
FAM	28.89% (11.41)	29.44% (15.41)	16.67% (34.43)	52.08% (34.36)
OMM	35.43% (17.29)	26.42% (12.17)	22.22% (34.30)	46.30% (30.55)
Combined groups	32.35% (14.97)	27.84% (13.66)	19.61% (33.95)	49.02% (32.03)
	Mindfulness meditation groups by word list order groups			
	AB word list order			
	True memory		False memory	
	Pre-meditation	Post-meditation	Pre-meditation	Post-meditation
FAM	29.38% (11.54)	33.58% (19.51)	29.63% (42.31)	51.85% (33.79)
OMM	36.11% (17.08)	32.50% (12.90)	45.83% (39.59)	50.0% (30.86)
Combined groups	32.55% (14.36)	33.07% (16.23)	37.25% (40.62)	50.98% (31.44)
	BA word list order			
	True memory		False memory	
	Pre-meditation	Post-meditation	Pre-meditation	Post-meditation
FAM	28.25% (12.15)	24.13% (5.20)	0% (0)	52.38% (37.80)
OMM	34.89% (18.36)	21.56% (9.55)	3.33% (10.54)	43.33% (31.62)
Combined groups	32.16% (16.0)	22.61% (7.94)	1.96% (8.08)	47.06% (33.46)

Data is presented as mean and standard deviation (SD). Note that false memories refer to recall of critical, non-studied word list items. Pre-meditation recall reflects performance for studied word lists that were not preceded by exposure to mindfulness meditation. Post-meditation recall, on the other hand, reflects performance for studied word lists that were preceded by single-session FAM or OMM. Deese-Roediger-McDermott (DRM) task list order AB group studied list A at pre-meditation and list B at post-meditation phases. List order BA group studied list B at pre-meditation and list A at post-meditation phases

### Correlation of MAAS Scores and Meditation Ratings with True and False Memory Recall

MAAS scores were not significantly correlated with recall percentages for true and false memory at pre- and post-meditation tests (all *p* > 0.091). Meditation Effort and Motivation VAS scores were not significantly correlated with true or false memory recall at pre- and post-meditation tests (all *p* > 0.06). Meditation Success VAS scores were not significantly correlated with false memory recall (all *p* > 0.22) or true memory recall at pre-meditation test (*p* = 0.52). However, there was a significant positive correlation between meditation Success VAS scores and true memory recall percentage at post-meditation, *r*(34) = 0.43, *p* = 0.011.

### Analysis of Non-studied or Critical Item Words

Groups did not significantly differ with respect to the count of recalled words not appearing on studied lists or representing critical items at pre-meditation recall test (Mean = 1.09, 95% CI: 0.79, 1.50, *p* = 0.85) or post-meditation recall test (Mean = 1.32, 95% CI: 0.67, 1.98, *p* = 0.192).

### Analysis of True and False Memory Recognition

Analysis of recognition accuracy for true and false memory words revealed a significant main effect of Memory Type, *F*(1,30) = 51.31, *p* < 0.001, *η*^*2*^_partial_ = 0.63. Recognition accuracy for true memory words (M = 63.14%, SE = 2.83) was significantly higher than for false memory words (M = 20.87%, SE = 3.88). No other significant main effects or interactions were found for recognition accuracy of true and false memory words (all *p* > 0.17). Analysis of recognition mean reaction time for true and false memory words revealed no significant main effects or interactions (all *p* > 0.19). No significant main effects or interactions for Group or List Order were observed in analysis of distractor word accuracy and mean reaction time (all *p* > 0.31).

### Correlation of MAAS Scores and Meditation Ratings with True and False Memory Recognition

Correlation analysis of MAAS scores and meditation Effort, Motivation and Success VAS ratings with recognition accuracy and mean reaction time revealed no significant correlations between MAAS scores or meditation Motivation VAS scores and recognition test performances (all *p* > 0.069). Meditation Effort VAS scores exhibited a significant positive correlation with recognition accuracy for post-meditation false memory words (critical items of post-meditation studies lists), *r*(34) = 0.36, *p* = 0.021. Meditation Success VAS scores had a significant positive correlation with recognition accuracy for post-meditation true memory words (*r*[34] = 0.42, *p* = 0.014) and a significant negative correlation with post-meditation false memory word recognition accuracy (*r*[34] =  − 0.38, *p* = 0.027).

## Discussion

Disparate findings exist in the literature regarding the influence of mindfulness meditation on true and false memory formation. Previous research has not accounted for distinct cognitive control states that arise from different mindfulness meditation styles (Lippelt et al., 2014). Thus, the present aim was to investigate if FAM and OMM states provide distinct influences on true and false memory. Based on the metacontrol state model (Hommel, 2015; Hommel & Colzato, 2017), it was predicted that increased cognitive control associated with FAM states would result in reduced false memory and increased true memory retrieval. As OMM states are thought to weaken the cognitive control (Hommel & Colzato, 2017; Lippelt et al., 2014), it was first hypothesised that OMM would lead to increased incidence of false memory.

### FAM and OMM Mindfulness States Might Increase False Memory

Contrary to the predictions, FAM and OMM did not differ in false memory recall at post-meditation. However, significant increase in false memory recall percentage between pre- and post-meditation tests suggests that both styles of mindfulness meditation might provide comparable influences on false memory formation. Single sessions of FAM and OMM might afford more generalised memory representations of encoded information (Schacter et al., 2011) either by heightening activation of semantically related information (Gallo, 2010; Gallo & Roediger, 2002; Roediger & McDermott, 1995) or strengthening gist representation (Brainerd & Reyna, 2002). Augmenting generalised memory through mindfulness states might explain wider benefits of mindfulness meditation on semantic memory tests, such as the verbal fluency task (Heeren et al., 2009; Zeidan et al., 2010).

Increased false memory recall following FAM and OMM mindfulness styles demonstrated in the present work is consistent with some of the previous studies investigating mindfulness meditation influences on false memory (Rosenstreich, 2016; Wilson et al., 2015). For instance, Wilson and colleagues (2015) reported a significant increase in false memories a single session of breath focused mindfulness meditation, which is very similar to the FAM technique employed in the present work. Increased false memory following OMM extends previous literature by demonstrating that mindfulness meditation influences on false memory might not be limited to FAM styles of meditation. To our awareness, this is the first demonstration of increased false memory following meditation states based on the OMM style alone. Previous demonstration of increased false memory following OMM has been when this style is combined with the FAM style in the meditation session (Calvillo et al., 2018; Meeks et al., 2019; Rosenstreich, 2016). The present findings illustrate that inclusion of the FAM style is not necessary to elicit false memory formation following single-session meditation. This can be elicited by the OMM style in of itself.

As with previous demonstrations, increased false memory following FAM and OMM styles was based on a single session of meditation. Meditation training might be necessary to elicit distinct influences of FAM and OMM styles on true and false memory formation in line with what might be predicted from the metacontrol state model (Hommel & Colzato, 2017) and empirical evidence of distinct cognitive control states established by these styles (Lippelt et al., 2014). Comparable effects of FAM and OMM styles on false memory demonstrated in the present experiment might be accounted for by meditation naïve participants’ reduced ability to achieve an OMM state and consequently, defaulting to a FAM state.

The present findings are in contrast with previous demonstrations of decreased false memory from mindfulness meditation or a lack of effect of mindfulness on false memory (Ayache et al., 2022; Baranski & Was, 2017; Sherman & Grange, 2020). Baranski & Was, (2017) explained that variable results could indicate that the effects of mindfulness meditation on false memory not robust and susceptible to several factors, such as participant’s degree of meditation training/exposure, mindfulness disposition, meditation session duration and the number of studied word lists. Studies reporting either a decrease in false memories (Baranski & Was, 2017) or no change in false memory recall or recognition at post-meditation (Sherman & Grange, 2020) did not control for prior meditation experience or cognitive training. Ayache et al. (2022), Rosenstreich & Ruderman (2017), and Rosenstreich (2016) previously controlled for prior meditation experience in their demonstrations of mindfulness meditation state effects on false memory. The rationale for this control being that formal meditation experience can increase dispositional mindfulness, which presents a potential confound in investigation of mindfulness state effects on false memory. We did not observe any significant correlation between mindfulness disposition and false memory recall indicating that such controls might not be necessary, and more widely, that mindfulness disposition is not a confounding factor in previous work. We also set out to account for potential individual differences on mindfulness state effects based on self-reported effort (Immink et al., 2017; Yamaya et al., 2023), success (Brandmeyer et al., 2019) and motivation (Spanos et al., 1980). We did not find any correlation of self-reported effort, success or motivation with false memory recall following mindfulness meditation. The present findings thus suggest that mindfulness meditation state effects on false memory recall do not depend on individual differences in perceived meditation efficacy or meditation motivation. However, it should be noted that recognition accuracy was dependent on individual differences in self-reported meditation effort and success. Meditation effort ratings were positively correlated with accurate rejection of false words as being previously studied following the meditation state. Ratings of meditation success were positively correlated with accurate recognition of list words presented after the meditation state. Thus, it is apparent that higher self-reported effort and success, which might reflect perceived efficacy in completing the mindfulness technique, are associated with heightened ability to distinguish true and false memory words in the recognition test. However, this explanation is complicated by the observation that higher self-reported success was also associated with poorer performance in rejecting false memory words associated with lists encoded after the meditation state. Further research is needed to address unclear relationships between self-reported meditation effort and success and recognition accuracy. Work is also needed to address why self-reported meditation measures were correlated with recognition accuracy but not memory recall. The present findings highlight that individual differences in perceived meditation effort and success need to be considered in research investigating meditation state effects on memory.

Mindfulness meditation influences on increased false memory reported here must be considered with some caution. First, the present demonstration does not include a control condition. Therefore, it remains plausible that the increased false memory recall observed at post-meditation is due to repeated encoding and retrieval tests, and not due to exposure to mindfulness meditation in the second round of encoding and retrieval. Previous work has demonstrated that practice effects are high for repeated memory tests (Benedict & Zgaljardic, 1998). Although alternate DRM lists were used at pre- and post-meditation phases, participants may have experienced increased false memory formation as a function of test-specific practice. That is, participants may have learned during the pre-meditation DRM phase that the lists followed a specific theme or gist (e.g. sleep) and applied this knowledge to the post-meditation learning phase. The absence of a control group prevents confident conclusions regarding the source of increased false memory, as these could be attributed to practice effects associated with the pre-/post-design. However, if the current findings were a consequence of practice, then an increase in true memory recall would also be expected. The current study did not detect such increase, raising some doubt for practice effects as an explanation for the present results. Nevertheless, future studies should compare FAM and OMM to a no-meditation, control group to ensure that increases in false memory formation are a function of the mindfulness meditation.

A second reason to treat the present demonstration of increased false memory following mindfulness meditation with caution lies in the possibility that the main effect of test time point might have been influenced by the order of word lists used in pre- and post-meditation conditions as illustrated in the complex three-way interaction based on list order, test time point and memory type factors. Indeed, it should be noted that, similarly to the lack of control in regard to previous meditation experience, research to date has also typically not controlled for previously established variables related to associative processing and the DRM lists in particular, including forward (Brainerd & Wright, 2005) and backward associative strength (Cann et al., 2011; Howe, Wimmer & Blease, 2009), and other constructs such as mean gist strength (Brainerd et al., 2020). Future work would be able to more thoroughly understand how meditation may influence false memory, and more important, which aspects and memory processes related to false memory, if such constructs are measured and accounted for. This is highlighted in the work of Howe, Wimmer and Blease (2009), who note that there exist differences in how false memories may be created between children and adults, potentially due to differences in inhibitory control and automaticity; this has implications for our understanding of false memory, and links the process to attentional control mechanisms potentially tagged by meditation. Thus, analyses such as the ones noted here may be important next steps in this literature.

### FAM and OMM Mindfulness States Do Not Influence True Memory

As FAM is thought to increase the cognitive control state, the second hypothesis predicted that this technique would lead to a greater increase in true memories than OMM relative to a pre-meditation control. This hypothesis was not supported, as FAM and OMM states did not differ with respect to true memory recall. Additionally, an increase in true memory formation from pre- to post-meditation was not detected for either group, indicating that whilst the cognitive control states induced by single sessions of FAM and OMM increase false memories, they do not influence true memory formation.

The current findings contradict previous reports of increased true memory following mindfulness meditation (Baranski & Was, 2017; Rosenstreich, 2016). This can be accounted for by differences in meditation experience provided before establishing the meditation state prior to memory encoding. For example, Rosenstreich (2016) trained participants in mindfulness meditation for 5 weeks as part of demonstrating increased true memory from mindfulness meditation. Basso et al., (2019) found that increases in true memory formation were evident after 8 but not 4 weeks of meditation training. Thus, in contrast to false memory, deriving increased true memory from single-session mindfulness meditation appears to rely on previous meditation training (Brown et al., 2016; Heeren et al., 2009; Lykins et al., 2012; Nyhus et al., 2019). The absence of increased true memory following mindfulness meditation demonstrated in the present work might be accounted for by the absence of meditation training in this sample.

That true memory, but not false memory, relies on previous mindfulness training can be explained by hierarchy of memory processing where generalised memories are more readily retrieved than perceptually or contextually detailed memories (Haque & Conway, 2010). Thus, smaller increments in attention control from single-session FAM and OMM in meditation novices might be sufficient to demonstrate immediate increases in low-level, semantically organised memory associated with false memory. Improvements in higher order memory processes contributing to true memory, on the other hand, might require a higher degree of attention control, which in turn requires more long-term structural or functional neural adaptations that rely on mindfulness meditation training (Lardone et al., 2018).

### Limitations and Future Directions

There are several potential reasons why the present study did not detect a significant difference between FAM and OMM on true and false memory formation. First, these techniques are not mutually exclusive, as OMM is recognised to involve aspects of FAM (Lee et al., 2018). Additionally, FAM is deemed suitable for beginners, whilst OMM is considered a more advanced technique (Lippelt et al., 2014). Consequently, the OMM group may have been influenced by a cognitive state more closely resembling that of FAM. Although FAM does not necessarily entail aspects of OMM techniques, participants in the FAM group may not have adhered to the specific instructions of their allocated meditation. Therefore, it cannot be confidently concluded that FAM and OMM groups achieved the distinct cognitive control states necessary to exert opposing influences on attention and subsequently memory. Due to differences in difficulty between FAM and OMM, future studies should consider training meditation-naïve participants in these techniques. Additionally, as FAM and OMM states have demonstrated differing patterns of neural activity (Yordanova et al., 2020), electroencephalography (EEG) may prove a useful tool for future research to objectively measure the extent to which participants are achieving these distinct states. In line with previous mindfulness meditation and false memory studies (Ayache et al., 2022; Sherman & Grange, 2020), future work should include manipulation checks to ensure that participants establish the intended distinct FAM and OMM states following encoding.

Secondly, the current project is limited by its small sample size which reduced the ability to detect a significant difference between groups. A post hoc power analysis revealed that the current study lacked sufficient power (0.41) to detect a significant difference between FAM and OMM groups at an alpha level of 0.05 and a moderate effect size (Faul et al., 2007). However, it should be noted that significant effects are argued to be reliable even under low statistical power conditions (Gelman & Carlin, 2014). Future research must recruit a much larger sample to ensure sufficient power to detect any potential differences between these techniques on true and false memory formation.

The current sample did include participants reporting insomnia, drug or alcohol dependence and diagnoses related to attention or cognitive impairments and psychiatric conditions. Our intention was to be inclusive of these participant characteristics given their presence in the general population. Nevertheless, the presence of these in the sample could have potentially influenced the results differently to what might be expected in a sample free from any cognitive, sleep, psychiatric or substance dependence conditions.

Another limitation of the present study was the lack of a controlled laboratory environment which prevented participant supervision and control over distracting input. Due to the COVID-19 pandemic, participants completed experiment conditions in their homes. Consequently, participants may have more readily disengaged from the meditation due to distraction, pervasive mind-wandering or boredom. As mind-wandering has been found to adversely affect cognitive performance (Zeidan et al., 2010), it is possible that participant disengagement, or lack of adherence to study protocol, may have influenced the results. The sample included participants who self-reported poor sleep quality, substance dependence, cognitive impairments or psychiatric conditions. Participants reporting these histories were not excluded to allow for an inclusive sample withing the age range. Nevertheless, there is potential that these participants might have differed with respect to how they completed the mindfulness techniques, as well as the DRM and retrieval tests.

Another limitation is that our analysis of recall percentage did not account for the potential influence of recall duration. The present recall tests did not control the amount of time available to recall items. Therefore, it is possible that whilst recall percentages did not differ between mindfulness meditation styles, the amount of time used to recall each item might have differed. Finally, the provocation of semantic-associative false memories using the DRM paradigm may not generalise to real-world false memories (Pardilla-Delgado & Payne, 2017; Zhu et al., 2013). Therefore, future research should investigate the influence of FAM and OMM states on different memory tasks that may better reflect real-world scenarios. To 2017, Ayache et al., (2022) alone have undertaken the most ecologically salient evaluation of mindfulness meditation influences on false memory using a virtual environment-based DRM task. The misinformation paradigm, whereby participants are exposed to an event, given misleading post-event information and are subsequently asked to recall the details, may be more generalisable to real-world forms of false memory (Nichols & Loftus, 2019). Thus, may help to elucidate the effects of FAM and OMM on everyday memory generalisation. Additionally, individual differences in attention have been linked to differences in the production of misinformation (Rivardo et al., 2011). Therefore, alternative false memory measures may engage separate attentional processes and thus may help to detect any potential differences between FAM and OMM states on true and false memory recall. Future work should continue to address whether individual differences in mindfulness disposition, which has been shown to moderate meditation influences on false memory (Ayache et al., 2022; Yeh & Lu, 2017) influences how FAM and OMM states influence false memory formation.

## Data Availability

Data available on request from the authors.
